# Neutrophil and Monocyte Bactericidal Responses to 10 Weeks of Low-Volume High-Intensity Interval or Moderate-Intensity Continuous Training in Sedentary Adults

**DOI:** 10.1155/2017/8148742

**Published:** 2017-06-01

**Authors:** David B. Bartlett, Sam O. Shepherd, Oliver J. Wilson, Ahmed M. Adlan, Anton J. M. Wagenmakers, Christopher S. Shaw, Janet M. Lord

**Affiliations:** ^1^MRC-ARUK Centre for Musculoskeletal Ageing Research, Institute of Inflammation and Ageing, University of Birmingham, Birmingham B15 2TT, UK; ^2^Duke Molecular Physiology Institute, Duke University School of Medicine, Durham, NC 27701, USA; ^3^Research Institute for Sport & Exercise Sciences, Liverpool John Moores University, Liverpool L3 3AF, UK; ^4^Carnegie School of Sport, Leeds Beckett University, Leeds L31 3HE, UK; ^5^School of Sport, Exercise and Rehabilitation Sciences, University of Birmingham, Birmingham B15 2TT, UK; ^6^Institute for Physical Activity and Nutrition, School of Exercise & Nutrition Sciences, Deakin University, Burwood, VIC, Australia

## Abstract

Neutrophils and monocytes are key components of the innate immune system that undergo age-associated declines in function. This study compared the impact of high-intensity interval training (HIIT) and moderate-intensity continuous training (MICT) on immune function in sedentary adults. Twenty-seven (43 ± 11 years) healthy sedentary adults were randomized into ten weeks of either a HIIT (>90% maximum heart rate) or MICT (70% maximum heart rate) group training program. Aerobic capacity (VO_2peak_), neutrophil and monocyte bacterial phagocytosis and oxidative burst, cell surface receptor expression, and systemic inflammation were measured before and after the training. Total exercise time commitment was 57% less for HIIT compared to that for MICT while both significantly improved VO_2peak_ similarly. Neutrophil phagocytosis and oxidative burst and monocyte phagocytosis and percentage of monocytes producing an oxidative burst were improved by training similarly in both groups. Expression of monocyte but not neutrophil CD16, TLR2, and TLR4 was reduced by training similarly in both groups. No differences in systemic inflammation were observed for training; however, leptin was reduced in the MICT group only. With similar immune-enhancing effects for HIIT compared to those for MICT at 50% of the time commitment, our results support HIIT as a time efficient exercise option to improve neutrophil and monocyte function.

## 1. Introduction

Neutrophils and monocytes are key components of the innate immune system and comprise the first line of defence against foreign pathogens [1, 2]. With falling birth rates and increasing longevity, we are an aging society with current demographic trends suggesting that 1 in 4 adults will be aged over 65 by 2050. Importantly, risk of infection is increased by an age-associated decline in neutrophil and monocyte function, which occurs as early as middle age [3, 4]. Key aspects of functional decline in neutrophils include reduced chemotaxis, phagocytosis, reactive oxygen species (ROS), and neutrophil extracellular trap (NET) production [3, 5, 6]. Similarly, dysfunctional monocytes are characterized by an altered phenotype including increased surface CD16 and impaired toll-like receptor (TLR) expression and function [7–9]. Furthermore, monocyte differentiation into macrophages is altered with a skewing towards a proinflammatory phenotype (M1) and reduced phagocytic capacity and antigen presentation [4]. Critically, dysfunctional immune responses are also associated with an elevated proinflammatory phenotype and likely contribute to age-related systemic inflammation, termed inflammaging, which increases the risk of several age-related chronic diseases [10, 11]. Identifying mechanisms to prevent or reverse neutrophil and monocyte dysfunction and reduce inflammaging is critical to improving immunity and reducing risk of infection and chronic disease as our population ages.

A growing body of evidence now suggests that exercise has systemic effects on immune function and inflammation. Although some of the anti-inflammatory effects of exercise can be attributed to the changes in adipose tissue, cellular immune function appears to be directly impacted also [12–14]. Our recent work suggests that neutrophil chemotaxis is better preserved in older adults who performed twice as many steps daily as age-matched controls [15]. Others have shown enhanced neutrophil phagocytosis and improved total numbers in relation to exercise training [16–18]. Exercise training has also been reported to influence monocyte function: CD16 expression and TLR expression were reduced; proinflammatory cytokine production was reduced; expression of the costimulatory molecule CD80 was increased [19–21]. Despite the evidence of an immune-modifying effect of exercise training, involvement in physical activity is low in the general population and is known to decline dramatically with age [22]; therefore, there is an urgent need to determine the optimal exercise exposure to benefit immune function.

As time constraints are considered a major barrier to exercise participation, novel exercise approaches in nonathletic sedentary populations have gained increasing attention [23]. High-intensity interval training (HIIT) offers an attractive approach by reducing the time commitment for exercise while providing cardiorespiratory fitness benefits similar to or greater than those of traditional moderate-intensity continuous training (MICT). Our group and others have shown significant improvements, comparable to MICT, in physiological, cardiometabolic, and psychological health with HIIT [24–27]. Although there is growing evidence that HIIT improves cardiometabolic health, there is less evidence to suggest that longitudinal HIIT has an effect on systemic inflammation and cellular immune function. Recently, Robinson and colleagues provided the first direct evidence that HIIT alters cellular immunity [20]. In this seminal paper, the authors demonstrated that 10 days of HIIT reduces monocyte, but not neutrophil, expression of TLR4, and lymphocyte expression of TLR2. The relevance of these changes to primary cellular functions is unclear. Furthermore, no studies have investigated the impact of a longer duration HIIT intervention on primary cellular immune function.

The purpose of our study was to determine the impact of ten weeks of group-based HIIT and MICT on neutrophil and monocyte function and systemic inflammation in sedentary healthy individuals. Specifically, we aimed to determine whether a shorter amount of exercise training time (HIIT) was comparable to a longer amount of exercise training (MICT). We examined neutrophil and monocyte phagocytosis and oxidative killing of *E. coli*; expression of CD16, TLR2, and TLR4; serum cytokine and specific hormone levels at baseline and following each intervention. We hypothesized that HIIT would be comparable to MICT in improving cellular immune function.

## 2. Methods

### 2.1. Participants and Experimental Procedures

Twenty-seven (43 ± 11 years) healthy (free of any known metabolic or cardiovascular disease and not taking any medication), inactive individuals were recruited for this substudy of a larger investigation examining the effects of HIIT on a range of cardiovascular, metabolic, psychological, and physical activity outcomes [24]. For experimental procedures including fitness testing, training procedures, body composition, and randomization, please refer to our previous publication [24]. Ethical approval was given by the University of Birmingham Research Ethics Committee, and all participants gave their written informed consent.

Briefly, aerobic capacity (VO_2peak_) was determined by a progressive exercise test to volitional exhaustion on an electronically braked cycle ergometer (Lode BV, Groningen, The Netherlands). Body composition was assessed using a single frequency bioimpedance device (Tanita BC 418 MA Segmental Body Composition Analyzer, Tanita, Japan). Participants were stratified into subgroups by age, gender, and body mass index (BMI) and randomly assigned to either HIIT or MICT.

All exercise sessions were conducted in a dedicated cycle suite at the University of Birmingham sports centre in groups of 10–15 participants. Training was carried out on commercial spinning bikes (Star Trac UK Ltd., Buckinghamshire, UK) and was led by a spin class instructor. Both groups were asked to attend three supervised sessions per week, with the MICT group prescribed two additional self-administered sessions for 10 weeks and participants instructed to achieve heart rate targets determined from the VO_2peak_ assessment. For both groups, each session was designed to be a group exercise-class design, which would differ slightly in duration or style depending on the day or week. As such, each person within groups completed similar programs and none were prescribed only one exercise duration per intervention. High-intensity interval training (HIIT) began with a 5-minute warm-up of low-intensity cycling before repeated high intensity sprints of between 15 and 60 seconds, interspersed with periods of active rest (45–120 seconds). Participants self-adjusted the resistance of the bikes to elicit a heart rate of >90% HR_max_ during the sprint intervals. Each HIIT session lasted 18–25 minutes. Moderate-intensity continuous training (MICT) began with a 5-minute warm-up of low intensity cycling before participants adjusted the resistance to elicit a heart rate of ~70% HR_max_. Each MICT session lasted 30–45 minutes; during the training period, participants were instructed to maintain their habitual dietary and physical activity patterns. As such, cumulative exercise exposure for each group amounted to 55 ± 10 minutes/week (HIIT) and 128 ± 44 minutes/week (MICT).

### 2.2. Blood Sampling

Participants arrived at the laboratory, having abstained from exercise in the previous 24 hours and at least 48 hours after their final exercise training. These times gave us confidence that any effects observed were for a training effect and not for an acute effect of the last session. Participants were seated for 15 minutes before peripheral venous blood samples were collected into vacutainers containing either heparin, EDTA, or a clotting agent for serum. Samples were then processed immediately for plasma and immune cell isolation while serum was left at room temperature for 30 minutes to clot. Serum and plasma were separated from blood by centrifugation (3000 ×g for 10 minutes), snap frozen in liquid nitrogen, and stored at −80°C until analysed.

### 2.3. Whole Blood Counts

Complete blood differentials, including leukocyte counts, were completed using EDTA-treated whole blood immediately after sampling on a fully automated Coulter™ ACT^diff^ haematology analyser (Beckman-Coulter, High Wycombe, UK). All samples were analysed in triplicate.

### 2.4. Mononuclear Cell Isolation

Peripheral blood mononuclear cells (PBMCs) were isolated from heparinised blood using density centrifugation. Briefly, blood was diluted 1 : 1 with phosphate-buffered saline (PBS) and layered over Ficoll-Paque™ PLUS (GE Healthcare, Uppsala, Sweden) at a blood Ficoll ratio of 4 : 3 mL. This was centrifuged at 400 ×g for 30 minutes at room temperature with no brake. Following centrifugation, mononuclear cells suspended at the interface of the Ficoll and plasma were removed and washed twice, 400 ×g for 10 minutes, in PBS. Cells were counted and viability assessed by Trypan Blue exclusion before being resuspended in PBS+ 1% bovine serum albumin (BSA, Sigma-Aldrich, Poole, UK) at 1 × 10^6^ cells·mL^−1^ and prepared for immunofluorescence staining.

### 2.5. Cell Surface Receptor Expression

Freshly isolated PBMCs were stained with combinations of anti-CD14-PcB (BD Bioscience, Oxford, UK, clone M5E2), anti-CD16-FITC (BD Bioscience, clone 3G8), anti-TLR2-APC (BD Bioscience, clone TL2.1), anti-TLR4-APC (Affymetrix eBioscience, Hatfield, UK, clone HTA-125), or their relevant concentration-matched isotype control for 30 minutes on ice in the dark. Following incubation, cells were washed in PBS/1% BSA and resuspended in PBS/1% BSA for analysis by flow cytometry. Mononuclear cells were identified by their typical forward versus side scatter, and 7000–10,000 CD14^+^ monocytes were acquired for analysis on a CyAn ADP™ 430 flow cytometer (Beckman-Coulter, High Wycombe, UK) and data were analysed using Summit v4.3 software (Dako, Cambridgeshire, UK).

For neutrophil surface phenotype, staining with the above antibodies, CD16, TLR2, and TLR4, was assessed in 100 *μ*L of whole blood. Blood was aliquoted into FACS tubes and placed on ice. Combinations of antibodies or isotype controls were added to blood and were incubated for 1 hour on ice in the dark. Following incubation blood was washed twice in PBS before adding 2 mL of 1x Fix/Lyse Buffer (Affymetrix eBioscience). Blood was placed at room temperature in the dark for 15 minutes to allow complete RBC lysis and fixation of WBCs. Following this, cells were washed twice in PBS and resuspended in PBS for analysis by flow cytometry. Granulocytes were gated by their typical forward versus side scatter, and 10,000 CD16^+^ neutrophils were acquired for analysis on a CyAn ADP 430 flow cytometer (Beckman-Coulter, High Wycombe, UK) and data were analysed using Summit v4.3 software (Dako, Cambridgeshire, UK).

### 2.6. Neutrophil and Monocyte Phagocytosis and Oxidative Burst

Phagocytosis and oxidative burst were assessed in whole blood using commercially available kits and manufacturers' guidelines (Phagotest and Phagoburst, BD Biosciences). Briefly, phagocytosis was assessed in heparin-treated whole blood and incubated at 4°C (control) or 37°C (test) with opsonised FITC-labelled *E. coli*. Phagocytosis was halted by the addition of cold phosphate-buffered saline (PBS) while cell surface-bound FITC was quenched by addition of Trypan Blue solution. Unbound-free bacteria were removed by washing in PBS and erythrocytes lysed and leukocytes fixed using 1% Fix/Lyse solution provided in the kit. Cell DNA was counterstained by an addition of propidium iodide (PI) before flow cytometry analysis was performed.

Oxidative burst was assessed in heparin-treated blood that was incubated at 37°C with opsonised *E. coli* (test) or PBS (control) for 10 minutes. Solution-containing dihyrdorodamine-123, which is converted to fluorescent rodamine-123 in the presence of reactive oxidants, was included for 10 minutes at 37°C. Oxidative burst was halted by the addition of erythrocyte lysis/leukocyte fixation buffer before leukocyte DNA was stained and flow cytometry analysis performed.

Phagocytosis and oxidative burst quantitation was performed on a CyAn ADP 430 flow cytometer equipped with three solid-state lasers. FITC and R-123 were detected in FL1 while PI was detected in FL2 using the Argon (405 nm) laser. 10,000 neutrophils and 5000–10,000 monocytes were acquired for analysis. Following compensation of FL1 versus FL2 phagocytic and oxidative burst was determined by the relative increase in median fluorescence intensity (MFI) in FL1 compared to negative controls. Data were analysed using Summit v4.3.

### 2.7. Serological Analyses

All measurements were made in duplicate using commercially available kits and manufacturer's guidelines. Serum interleukin- (IL-) 1*β*, IL-4, IL-6, IL-8, IL-10, IL-13, IL-17, granulocyte/macrophage colony-stimulating factor (GM-CSF), and tumor necrosis factor (TNF)*α* were simultaneously measured by multiplex luminometry (Bio-Rad, Hemel Hempstead, UK). Samples we0re analysed using a Bio-Plex Luminex^200^ platform equipped with a 635 nm red and 532 nm green laser using Bio-Plex Manager™ software. Detection of C-reactive protein (CRP) was by high-sensitivity enzyme-linked immunosorbent assay (ELISA) using a commercial kit (IBL International, Hamburg, Germany). Plasma cortisol and DHEAs were assessed separately by ELISA (IBL International, Hamburg, Germany) and plasma adiponectin and leptin were assessed separately by solid phase sandwich ELISA (R&D Systems, Abingdon, UK).

### 2.8. Statistical Analysis

Statistical analysis was conducted using PASW version 18.0 (Chicago, IL, USA), and data are presented as mean ± SD unless otherwise stated. Normality was assessed using Kolmogrov-Smirnov analysis; natural log transformation of distributed variables violating normality was completed. Data were analysed using repeated measures ANOVA to assess the effect of training on immune function and interactions with training × exercise group. Age and body fat percentage were included as covariates due to the influence of age on immune function and inflammation, and the small changes in body fat were observed in the MICT group. Statistical significance was accepted at *p* < 0.05.

## 3. Results

### 3.1. Participant Characteristics, Exercise Capacity, and Body Composition

Body composition and aerobic capacity measures are presented in Table 1. No differences were detected between the groups at baseline (*p* > 0.05). As with the larger study, the HIIT group completed on average 57% less total training time commitment compared to the MICT group (*p* < 0.001). There were significant main effects of training for VO_2peak_ [F(1,25) = 49.6; *p* < 0.001; *η*_p_^2^ = .67] with increases postexercise of 9% for both HIIT and MICT (both *p* < 0.001). Neither body mass nor BMI (both *p* > 0.05) was reduced by training in this cohort. However, there were significant main effects of training for body fat percentage [F(1,25) = 7.9; *p* = 0.01; η_p_^2^ = .27], with reductions observed for MICT (*p* = 0.04) but not HIIT (*p* = 0.08). No differences between groups for effects of training were observed.

### 3.2. Immune Responses

A primary mechanism of bacterial clearance by neutrophils and monocytes is an ingestion of microbes through phagocytosis and subsequent killing in the phagolysosome as a result of exposure to reactive oxygen species.  Table 2 shows the neutrophil and monocyte bactericidal capacity following HIIT or MICT.

All neutrophils (100%) from subjects in both groups ingested opsonized *E. coli* and produced an oxidative burst. There were significant main effects of training for the amount (MFI) of *E. coli* ingested by neutrophils [F(1,25) = 7.5; *p* = 0.011; *η*_p_^2^ = .24], with increases observed for HIIT (*p* = 0.023) and MICT (*p* = 0.049). There were also significant main effects of training for the amount (MFI) of ROS produced against *E. coli* by neutrophils [F(1,25) = 12.2; *p* = 0.002; *η*_p_^2^ = .36], with increases observed for HIIT (*p* = 0.03) and MICT (*p* = 0.004).

Similarly, there were significant main effects of training for the amount (MFI) of *E. coli* ingested by monocytes [F(1,25) = 18.7; *p* < 0.001; *η*_p_^2^ = .46], with increases observed for HIIT (*p* = 0.005) and MICT (*p* = 0.002). Although there were no increases in monocyte oxidative burst (*p* > 0.05), there were significant main effects of training for the percentage of monocytes producing an oxidative burst [F(1,25) = 11.1; *p* = 0.003; *η*_p_^2^ = .33], with increases observed for HIIT (*p* = 0.03) and MICT (*p* = 0.006).

**Figure 1:**
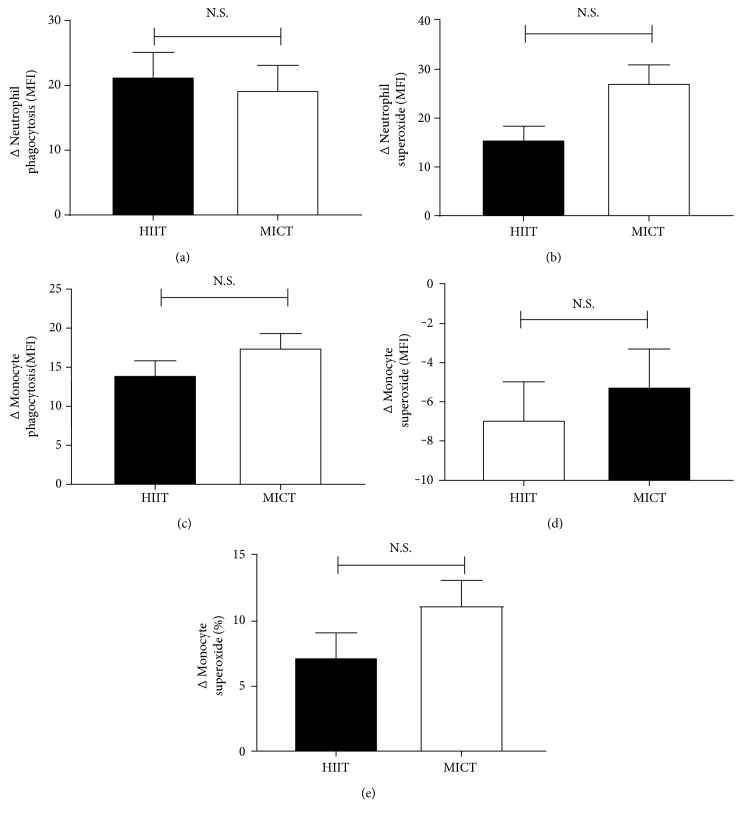
Final minus baseline change scores for group immune function. HIIT was comparable to MICT for changes in neutrophil phagocytosis (a), neutrophil superoxide (b), monocyte phagocytosis (c), monocyte superoxide (d), and percentage of monocytes-producing superoxide (e). N.S.—nonsignificant differences between groups.

There were no differences between groups for any of the functional measures assessed suggesting both routines are equally effective (Figure 1), though there was a trend for MICT to have greater improvements in neutrophil superoxide production (Figure 1(b), *p* = 0.094).

In order to determine whether changes in cell function reflect changes in total cell numbers, Table 3 shows complete blood differentials before and after training. There were no effects of training on total white blood cell (WBC), lymphocyte, neutrophil, or monocyte counts (all *p* > 0.05). To better understand the changes in cellular function observed, we assessed expression of CD16, TLR2, and TLR4 on monocytes and neutrophils. No effect of exercise was observed for TLR2, TLR4, or CD16 on neutrophils (data not shown; *p* > 0.05 for all). There were significant main effects of training on the percentages of monocyte subsets. Training increased the percentage of CD14^+^/CD16^−^ [F(1,25) = 11.3; *p* = 0.004; *η*_p_^2^ = .31] with increases observed for HIIT (*p* = 0.008) and MICT (*p* = 0.024). Reductions were observed for the percentage of CD14^+^/CD16^int^ monocytes in both groups (*p* < 0.05). Reductions in the percentage of CD14^+^/CD16^bright^ monocytes were observed in the HIIT (*p* = 0.031) group but not in the MICT (*p* = 0.071). There were significant main effects of training for expression of TLR4 on CD14^+^/CD16^bright^ monocytes [F(1,25) = 16.54; *p* < 0.001, *η*_p_^2^ = .26] with reduced expression observed for HIIT (*p* = 0.001) and MICT (*p* = 0.001). TLR2 expression was higher at all times on CD14^+^/CD16^−^ and CD14^+^/CD16^int^ compared to CD14^+^/CD16^bright^ populations (*p* < 0.05). There were significant main effects of training for expression of TLR2 on CD14^+^/CD16^int^ monocytes [F(1,25) = 19.42; *p* < 0.001, *η*_p_^2^ = .20] with reduced expression observed for HIIT (*p* < 0.001) and MICT (*p* = 0.001). No differences for training between groups were observed. There were no differences within or between groups for surface expression of TLR2 or TLR4 on CD16^+^ neutrophils (data not shown).

### 3.3. Serological Analysis

Exposure to systemic inflammation, neuroendocrine hormones, and metabolic hormones can influence immune cell function. However, no differences were observed for any inflammatory cytokines or acute phase proteins or endocrine hormones at baseline or in response to exercise training, Table 4 (all *p* > 0.05). There were significant main effects of training for the metabolic hormone leptin [F(1,25) = 6.9; *p* = 0.014; *η*_p_^2^ = .22] with reductions in the MICT group (*p* = 0.043) but not in the HIIT group (*p* = 0.127).

## 4. Discussion

This study shows that, in sedentary men and women, ten weeks of low-volume high-intensity interval training was comparable to moderate-intensity continuous training at improving neutrophil and monocyte bactericidal capacity while reducing CD16, TLR2, and TLR4 on CD14^+^ monocytes but not neutrophils. Systemic inflammation and endocrine responses were unaffected by either of the training interventions, although leptin was lower following MICT which was associated with a reduced body fat percentage. As previously reported in our primary study, VO_2peak_ was significantly improved and body fat percentage was marginally reduced in MICT with no difference observed between groups [24]. Critically, no differences between groups were observed for our immunological analyses suggesting that for half the prescribed exercise time, HIIT can improve immune function to a similar extent as MICT. Therefore, we suggest that HIIT might be an effective means to improve fitness and immune function in populations who find typically prescribed continuous exercise difficult.

### 4.1. Changes in Immune Cell Function

Although changes in primary immune cell function are associated with risk of infectious episodes, surprisingly, little is known about exercise training and neutrophil and monocyte bactericidal functions. Both cells are central to the early resolution of infection, primarily by phagocytosis of the pathogen and oxidative killing of the pathogen.

When compared to sedentary healthy matched controls, physically active individuals aged between 20 and 60 years have increased neutrophil phagocytosis and ROS production [16, 18]. Neutrophil phagocytosis was also improved following 2 months of moderate-intensity exercise training in middle-aged healthy men [28]. Our data adds to this literature in suggesting that HIIT and MICT are equally capable of improving neutrophil bactericidal capacity and likely reducing risk of infectious episodes.

It is still unclear how these functional improvements occur. We found no effect of training on neutrophil expression of TLR4, the primary TLR responsible for recognition of the lipopolysaccharide (LPS) membrane component of *E. coli*. Although Robinson and colleagues did not assess neutrophil bactericidal capacity, expression of TLR4 was reduced following 10 days of MICT but not HIIT [20]. It is unclear why we did not observe a similar effect or whether reduced TLR4 would in fact reduce bactericidal function. Our study and theirs assessed immune function greater than 48 hours after the last exercise session, and so, we are confident it is not due to blood draw timing. One potential explanation is that acute bouts of exercise are capable of selective clearance of dysfunctional immune cells [29]. If exercise clears dysfunctional neutrophils from the system acutely, improving the functionality of the remaining pool in the longer term, then the 10-week intervention used here could benefit from this change and explain why we saw improved neutrophil function.

We have previously shown that habitual physical activity (~10,000 versus ~5000 steps/day) is associated with enhanced neutrophil functions in the absence of surface receptor differences [15]. Our results in this cohort also suggest that neutrophil CD16 expression is not influenced by exercise training. Our lack of change in TLR and CD16 expression suggests that there are instead intrinsic cell signalling alterations associated with improved neutrophil function. Neutrophil bactericidal functions are regulated by several signalling pathways, downregulation of which can compromise mechanism such as phagocytosis and oxidative burst. Such effects are seen with aging, with reduced MAP kinase amongst the differences seen in neutrophils from older donors [30]. To our knowledge, there are no reports of the effects of exercise on MAP kinase signalling in immune cells, though this has been reported in skeletal muscle [31]. Future research should aim to determine neutrophil signalling pathways influenced by exercise training in order to understand exercise-mediated mechanisms.

With a progressive age-associated decline in neutrophil function, there is an enhanced risk of infection. As neutrophils are the first line of defence against pathogenic invasion, they are integral to the effective resolution of infection. Although our participants were aged on average 43 years old (23–60 years), our results indicate a potential route to improving neutrophil function in at risk groups. To date, no study has assessed the impact of HIIT on neutrophil function in over 60-year olds. Future studies should aim to assess immune functional responses to HIIT in populations showing clear signs of immunosenescence in order to determine whether it is possible to reverse or delay immunosenescence. Critically, our data highlights that neutrophil bactericidal capacity can be improved by higher intensity exercise with significantly less time commitment.

Monocytes make up a relatively small proportion of circulating leukocytes (2–12%). However, due to their diverse role in immune function and inflammation, they have received more interest than neutrophils in the exercise literature. The majority of literature is focused on monocyte phenotype and proinflammatory cytokine production and less on bactericidal activity [32–34]. We show for the first time that monocyte bactericidal function is improved by 10 weeks of HIIT. We observed a significant improvement in phagocytic capacity in both exercise groups. Although we did not see more ROS production on a per cell basis, the percentage of ROS-producing monocytes was significantly increased. It is unclear how these improvements were achieved. The percentage of cells producing ROS suggests that not all monocytes were equally phagocytically active, and our results may be influenced by the training-induced increase in CD16-negative monocytes (see below) which could influence ROS production. Schaun and colleagues observed no difference in monocyte phagocytosis following 12 weeks of aerobic exercise training [35], though they used a TLR2 agonist (zymosan) rather than bacteria. Of interest is that basal and stimulated monocyte ROS production and phagocytosis is suggested to be higher in obese individuals [36]. However, we saw no association with weight and ROS production or phagocytosis and as there was minimal weight loss, we can assume that increased phagocytosis was not influenced by changes in body fat.

Research on monocyte function and phenotype has focused on acute single session or short-term (days) bouts of exercise. The Nieman group have pioneered monocyte function research over the last twenty-years and have shown interesting acute effects of exercise [37–39]. Acute exercise is associated with an intensity-dependent increase in numbers of circulating monocytes that are predominated by a proinflammatory phenotype [40–42]. Monocyte phagocytosis is transiently increased by acute exercise and is associated with the degree of inflammatory response to exercise [43]. However, Nieman and colleagues also recently showed that monocyte bactericidal capacity is diminished in response to overtraining, muscle damage and elevated inflammation [37]. Although we did not assess acute exercise-mediated immune responses, it is likely that each exercise session resulted in transient changes in function. We can only assume that each exercise bout has a small but significant impact on basal function, progressively improving it over time.

We observed significant changes in monocyte phenotype following exercise training, suggesting an altered inflammatory potential. Specifically, there were reductions in the cell surface expression of CD16. CD16 is typically associated with a proinflammatory subtype with higher basal and stimulated production of cytokines such as TNF*α* [44]. Furthermore, we observed significant reductions of TLR4 expression on intermediate and TLR2 on nonclassical CD16^+^ proinflammatory monocytes. Our work is in agreement with Robinson and colleagues who showed that 2 weeks of HIIT was sufficient to reduce monocyte expression of TLR4. Although their study did not show reduced TLR2 on monocytes, they did find an effect on lymphocytes suggesting that TLR2 is influenced by training and monocyte expression may require more or less training time [20].

As with our neutrophil data, we did not observe functional monocyte improvements associated with relevant changes in cell surface receptor expression (i.e., more phagocytosis with more TLR4). Therefore, functional improvements are likely associated with intrinsic cell signalling changes similar to what we suggest in neutrophils. Recent gene expression analysis of acute interval exercise bouts suggests that monocytes may be directed towards an anti-inflammatory profile with downregulation of TNF, TLR4, and CD36 genes [41]. Additionally, metabolic disorders such as type 2 diabetes and obesity are associated with increased TLR expression and activation [45]. Although our participants were metabolically healthy, glucose tolerance and insulin sensitivity were improved following training suggesting a link with metabolic control and TLR expression [24]. Taken together, HIIT has the potential to modify monocyte proinflammatory phenotype and contributes to improved bactericidal capacity. Future research should attempt to determine the acute and chronic mechanisms by which exercise influences inflammatory monocyte functions.

### 4.2. Impact of HIIT on Serological Measures

Although we observed altered cellular responses to training which can be associated with an anti-inflammatory effect, there were no reductions in serological markers of inflammation. The anti-inflammatory effects of exercise have been extensively researched and reviewed [12, 46]. However, it remains unclear what the inflammatory response is in the absence of weight loss and what the consequences of this might be [47]. Acute exercise is associated with both an immune cell redistribution and an inflammatory response which following exercise cessation returns to normal levels between 0.5 and 24 hours [48–50].

These effects have led many to conclude that exercise training has a role in controlling chronic low-grade inflammation. However, it is becoming clear that low-grade inflammation may be influenced less by exercise training and more by weight loss [20, 32]. When accounting for weight change in the analysis of exercise-mediated effects on inflammation markers such as CRP, effects of exercise are often lost [32, 51]. In agreement with these reports, we observed no changes in concentrations of a number of basal inflammatory cytokines or acute phase proteins. Similarly, Robinson and colleagues also found no significant effects of HIIT or MICT on systemic inflammation [20]. A number of recent studies have highlighted the acute inflammatory response to HIIT and its similarity to MICT; however, these snapshots do not inform of longitudinal responses [50, 52]. As inflammatory biomarkers are used more in prediction of disease outcomes, future research should aim to determine the discrete and analogous effects of intervention-specific weight and fitness responses on inflammation.

We did find that MICT but not HIIT was associated with a small but significant 1.4% reduction in body fat. This was aligned with a significant 10% reduction in the adipose tissue-derived adipokine leptin. Although leptin inhibits hunger, it can have proinflammatory effects on immune cells [53]. Whether the reduction in leptin was directly associated with immune function remains unclear; however, it is unlikely to be influential on systemic inflammatory responses.

With a growing body of evidence suggesting links between the endocrine system, systemic inflammation, and physical activity, we measured cortisol and DHEAs [11, 54–56]. Although others have shown exercise effects for cortisol and DHEAs with reductions in cortisol [57], we saw no effect in the present study. These findings are not surprising in light of the lack of changes observed for inflammatory cytokine measures. Taken together, our results are in agreement with other exercise studies showing that exercise training with minimal weight change is not associated with reduced systemic inflammation.

### 4.3. Limitations

Our study is not without limitations. We have previously described the major study limitations and, here, will describe the substudy-specific limitations [24]. The high proportion of women may have influenced our findings. We were unable to control for menstrual cycle during our measurements and as such, women were likely at different stages of menses during the study measurements. It is unclear the interactions of the menstrual cycle, exercise training, and immune function in our study. Others have suggested that the menstrual cycle during acute exercise may be associated with altered mucosal immunity and may transiently alter subsets of lymphocytes [58, 59]. Although we are unaware of previous studies assessing menstrual cycle interactions with exercise training and neutrophil or monocyte bactericidal function, we cannot discount a possible influence. Future studies should aim to assess the role of the menstrual cycle in relation to exercise and immune function. Although previous studies have shown gender differences in response to HIIT, these are often focused on metabolic control including glycogen breakdown and insulin sensitivity [60]. Although we did not have statistical power to analyse gender differences, the results of female participants were the same as when combined with men.

Our study was designed to determine whether a shorter amount of exercise training time, at a higher intensity, was comparable to a longer amount of exercise time, at a lower intensity for key outcome measures. The groups differed on total exercise time per session, with HIIT performing 18–25 minutes/session while MICT performed 30–45 minutes/session. HIIT participants performed on average 2.6 sessions/week, while MICT participants performed on average 3.4 sessions/week. Cumulative exercise time per week was 55 ± 10 minutes (HIIT) versus 128 ± 44 minutes (MICT). As such, these differences for time present a technical limitation, which prevents us from determining a mechanistic role for exercise intensity. Although this was not our aim, had we controlled for exercise time, the results such as VO_2_ would likely have been different. Given that HIIT was 57% less time-consuming than MICT and provided similar benefits, it could be surmised that longer durations of HIIT would provide greater benefits than MICT. However, the feasibility of these remains unknown and it might be that adherence would drop from 82%.

In addition to time limitations, we also did not control for and were unable to assess energy expenditure between the groups. Although HIIT might have had higher energy expenditure per session, it is likely that the MICT group had greater overall energy expenditure as evident by the small but significant reduction in body fat percentage in the MICT group. The HIIT group, although not significant, had a trend towards reduced body fat percent. Both groups were advised not to alter their diet or current physical activity levels. We did not specifically control for diet and as such, we are uncertain whether participants changed their diet that resulted in a reduced body fat percentage in the MICT group. Because of this small change, we cannot discount that the effects seen in MICT were driven by changes in body fat. However if this is the case, then the effects of HIIT and MICT are through different pathways, one adipose and the other fitness mediated. It is likely that small changes in fitness and small changes in adipose tissue synergize to result in larger changes in other organs. Until exercise studies can discriminate between fat and fitness, we will be unable to give specific cause and effects. However, HIIT might be a useful model to start teasing out these differences in at risk populations.

## 5. Conclusion

In summary, this study is the first to demonstrate in sedentary adults an immune bactericidal enhancing effect of HIIT. As these adaptations were comparable to those following MICT despite less than 57% of the total exercise time, these results support HIIT as a time-efficient exercise option to improve neutrophil and monocyte function. Furthermore, as previously reported, HIIT had better adherence than MICT (82 ± 14% versus 62 ± 13%, resp.) [24]. Although time commitment was reduced, it is unclear what total workload performed was for each group. Because both groups improved physiological fitness and immunological parameters similarly, it is likely that total work performed was similar between the groups. As such, for those considering HIIT as an exercise program, reduced time does not equate to less work. The same amount of work must be performed, just in less amount of time. Therefore, reduced time commitment and higher adherence combined with the social aspect of group HIIT training could provide an effective means to engaging older adults, most in need of immune function improvements, in exercise training.

Our findings support the proposal that improved function of innate immune cells is a potential anti-immunosenescence response to exercise training of high- or moderate-intensity exercise training. Both HIIT and MICT altered monocyte, but not neutrophil, expression of key surface receptors suggesting that functional improvements are related to intrinsic cellular signalling pathway changes. Neutrophils and monocytes are key intermediaries in the resolution of infection, tissue repair, and control of chronic systemic inflammation. Their age-associated functional decline is central to the development of many age-related diseases including cardiovascular disease and metabolic disease. Future research should aim to determine if the cellular immune responses to exercise training are associated with altered intrinsic cellular signalling and whether this translates directly to improved cardiometabolic health, reduced disease risk, and reduced risk of infection in at risk populations.

## Figures and Tables

**Table 1:** Group baseline and final values for participant body composition and fitness measures.

	HIIT		MICT	
	Baseline	Final	Baseline	Final
*n* (males/females)	14 (4/10)		13 (5/8)	
Age (years)	42 (12)		45 (10)	
Body composition				
Height (cm)	167 (7)		166 (9)	
Body mass (kg)	78.1 (19.9)	77.7 (19.4)	79.0 (15.2)	78.4 (16.2)
BMI (kg/m^2^)	28.1 (6.1)	27.9 (6.0)	28.1 (4.7)	27.9 (5.0)
Fat mass (%)	32.9 (8.2)	31.8 (8.2)	32.2 (8.6)	30.9 (8.7)^∗^
Physical fitness				
VO_2max_ (L min^−1^)	2.4 (0.6)	2.7 (0.7)^∗∗∗^	2.6 (0.6)	2.9 (0.7)^∗∗∗^
VO_2max_ (mL/kg min^−1^)	31.5 (6.4)	35.0 (8.4)^∗∗∗^	32.6 (5.8)	35.8 (7.1)^∗∗∗^

BMI: body mass index. Data are mean (SD) unless otherwise stated. ^∗^*p* < 0.05, ^∗∗∗^*p* < 0.001 are significant within group change scores.

**Table 2:** Group baseline and final values for neutrophil and monocyte phagocytosis of *E. coli* and oxidative burst towards *E. coli.*

	HIT		MICT	
	Baseline	Final	Baseline	Final
Neutrophil				
Phagocytosis (MFI)	130.6 (16.6)	152 (17.2)^∗^	126.2 (12.5)	145.5 (14.1)^∗^
Oxidative burst (MFI)	69.0 (24.5)	74.6 (22.1)^∗^	77.6 (18.2)	104.8 (16.5)^∗∗^
Monocyte				
Phagocytosis (MFI)	99.3 (10.4)	113.3 (11.6)^∗∗^	92.9 (12.2)	110.4 (13.8)^∗∗^
Oxidative burst (MFI)	27.5 (8.4)	22.7 (9.1)	30.9 (11.7)	27.8 (13.5)
Oxidative burst (%)	74.4 (10.0)	81.6 (11.1)^∗^	74.4 (13.7)	85.6 (12.1)^∗∗^

MFI: median fluorescence intensity. Data are mean (SD). ^∗^*p* < 0.05 and ^∗∗^*p* < 0.01 are significant within group change scores.

**Table 3:** Group baseline and final values for total numbers of white blood cells, percentages of monocyte subsets, and expression of TLR4 and TLR2.

	HIIT		MICT	
	Baseline	Final	Baseline	Final
Total WBC (×10^9^ L^−1^)	7.4 (2.7)	8.2 (2.1)	8.2 (1.1)	7.7 (2.4)
Lymphocytes (×10^9^ L^−1^)	2.4 (0.9)	2.5 (0.7)	2.5 (0.4)	2.7 (0.5)
Neutrophils (×10^9^ L^−1^)	4.6 (1.8)	5.2 (1.7)	5.2 (1.2)	4.4 (2.0)
Monocytes (×10^9^ L^−1^)	0.3 (0.2)	0.4 (0.2)	0.4 (0.2)	0.5 (1.1)
CD14^+^/CD16^−^ (%)	87.2 (4.7)	91.1 (4.5)^∗∗^	86.9 (5.8)	89.8 (6.1)^∗^
TLR4 (MFI)	3.9 (1.2)	3.8 (1.4)	3.9 (1.2)	3.8 (1.3)
TLR2 (MFI)	119 (11.2)	120 (10.1)	121 (13.4)	123 (14.1)
CD14^+^/CD16^int^ (%)	4.4 (2.8)	3.1 (2.2)^∗^	4.7 (2.4)	3.6 (2.2)^∗^
TLR4 (MFI)	5.0 (2.1)	4.9 (2.8)	5.1 (2.0)	4.9 (2.1)
TLR2 (MFI)	123 (15.3)	116 (16.5)^∗^	124 (18.9)	119 (19.9)^∗^
CD14^+^/CD16^bright^ (%)	8.1 (4.4)	5.8 (3.9)^∗^	8.4 (3.1)	6.9 (4.2)
TLR4 (MFI)	4.6 (3.2)	4.0 (2.7)^∗∗^	4.5 (3.0)	3.9 (2.7)^∗∗^
TLR2 (MFI)	86 (10.9)	85 (12.6)	91 (13.2)	93 (14.0)

WBC: white blood cell; CD: cluster of differentiation; TLR: toll-like receptor; MFI: median fluorescence intensity. Data are mean (SD). ^∗^*p* < 0.05 and ^∗∗^*p* < 0.01 are significant within group change scores.

**Table 4:** Group baseline and final values for inflammatory cytokines and acute phase proteins, endocrine, and metabolic hormones.

	HIIT		MICT	
	Baseline	Final	Baseline	Final
Proinflammatory				
IL-1*β* (pg mL^−1^)^a^	0.1 (0.08)	0.1 (0.1)	0.5 (1.2)	0.4 (0.9)
IL-6 (pg mL^−1^)^a^	1.4 (1.6)	1.0 (1.0)	1.2 (1.0)	0.8 (0.8)
IL-8 (pg mL^−1^)	5.7 (2.1)	4.4 (1.5)	5.7 (2.5)	5.4 (2.3)
IL-17 (pg mL^−1^)^a^	5.5 (17.9)	5.2 (15.7)	13.8 (23.4)	5.5 (14.7)
TNF*α* (pg mL^−1^)^a^	0.8 (2.1)	0.1 (0.4)	ND	0.2 (0.5)
CRP (mg L^−1^)^a^	3.0 (4.3)	2.1 (2.2)	1.2 (1.4)	1.6 (1.8)
GM-CSF (pg mL ^−1^)^a^	0.02 (0.06)	ND	0.02 (0.07)	0.02 (0.07)
Anti-inflammatory				
IL-4 (pg mL^−1^)^a^	0.04 (0.1)	0.06 (0.1)	0.02 (0.05)	0.05 (0.07)
IL-10 (pg mL^−1^)^a^	0.3 (1.1)	ND	ND	ND
IL-13 (pg mL^−1^)^a^	0.7 (1.2)	0.6 (0.9)	1.1 (1.8)	0.7 (1.1)
Endocrine				
Cortisol (nmol L^−1^)^a^	200 (61.3)	190 (62.1)	197 (98.6)	183 (56.0)
DHEAs (nmol L^−1^)	3563 (1965)	3841 (2087)	4464 (2335)	4144 (1739)
Cortisol: DHEAs	0.07 (0.03)	0.06 (0.04)	0.05 (0.03)	0.05 (0.02)
Metabolic				
Adiponectin (*μ*g mL^−1^)	3.8 (1.9)	3.6 (1.8)	3.3 (1.2)	3.1 (1.3)
Leptin (ng mL^−1^)	19.3 (11.3)	18.0 (10.8)	16.4 (9.6)	14.6 (10.2)^∗^
Leptin: adiponectin	6.0 (4.4)	6.1 (4.3)	6.0 (4.6)	5.8 (5.3)

IL: interleukin; TNF: tumour necrosis factor; ND: nondetectable; CRP: high sensitivity C-reactive protein; GM-CSF: granulocyte/macrophage colony-stimulating factor; DHEAs: dehydroepiandrosterone sulphate. Data are mean (SD). ^a^Not normally distributed and were log transformed. ^∗^*p* < 0.05 significant within group change scores.
